# Endothelial Glycocalyx Protection in Sepsis

**DOI:** 10.14789/jmj.JMJ23-0041-P

**Published:** 2024-02-10

**Authors:** JERROLD H LEVY, TOSHIAKI IBA

**Affiliations:** 1 Department of Anesthesiology, Critical Care, and Surgery, Duke University School of Medicine, Durham, NC, USA; 1 Department of Anesthesiology, Critical Care, and Surgery, Duke University School of Medicine, Durham, NC, USA; 2 Department of Emergency and Disaster Medicine, Juntendo University Graduate School of Medicine, Tokyo, Japan; 1 Department of Emergency and Disaster Medicine, Juntendo University Graduate School of Medicine, Tokyo, Japan

**Keywords:** glycocalyx, endothelial cell, sepsis, shock, anticoagulation, syndecan

## Abstract

The glycocalyx serves as the covering layer of the luminal surface of vascular endothelial cells, comprising proteoglycans, glycosaminoglycans, and adherent plasma proteins. This intricate structure is crucial in promoting antithrombogenicity, controlling vascular permeability, regulating vascular tone, and managing leukocyte/platelet adhesion. However, during sepsis, the glycocalyx undergoes significant degradation through inflammatory mechanisms; this process can be further facilitated by treatment for sepsis and septic shock. Therefore, it is crucial to exercise careful management to avoid damage to the glycocalyx during sepsis treatment.

The vascular endothelial surface is covered by a gel-like layer, namely glycocalyx. The glycocalyx is composed of membrane-binding proteoglycans (syndecan and glypican), glycosaminoglycan side-chains (heparan sulfate and chondroitin sulfate) conjugated with the core protein of the proteoglycans, and high-molecular-weight polysaccharide hyaluronan that interacts to transmembrane receptor CD44. The hydrophilic polysaccharides bind functional proteins such as albumin and antithrombin^1)^ (Figure 1). The vascular glycocalyx exhibits important roles that include attenuating flow resistance, controlling vascular tone, maintaining antithrombogenicity, and regulating vascular permeability. However, since the glycocalyx is highly fragile and easily damaged via upregulated inflammatory reactions, increased shedders such as metalloproteinases, heparanase, and hyaluronidase are needed. Intravascular inflammation is closely associated with glycocalyx degradation, leading to vascular permeability, edema formation, thrombotic shift, and neutrophil and platelet adhesion^1)^. Consequently, the circulating glycocalyx components, such as syndecan and hyaluronic acid, are degraded and serve as biomarkers of endothelial damage^2)^. The derangement of glycocalyx is tightly connected with the presence of organ dysfunction and high mortality in sepsis^3)^. Therefore, protection and maintenance of glycocalyx are critical in sepsis management. The following part introduces the potential pragmatic approach for glycocalyx protection.

**Figure 1:**
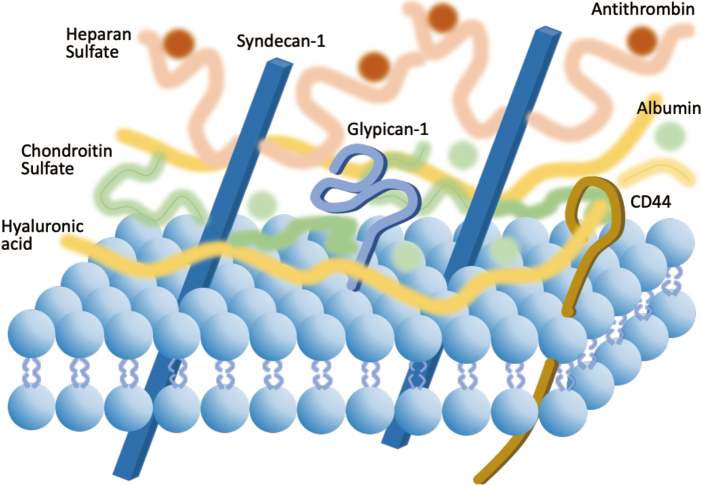
The structure of the endothelial glycocalyx The major components of the glycocalyx are transmembrane glycoprotein i.e., syndecans, membrane-attached glypicans, and hydrophilic polysaccharides, including heparan sulfate, chondroitin sulfate, and hyaluronan. Physiological plasma proteins, including albumin and antithrombin, are maintained in this structure.

Hypervolemia is thought to be associated with increased glycocalyx degradation. Previous studies suggested that hypervolemia induces atrial natriuretic peptide release, leading to glycocalyx degradation^4)^. A randomized controlled trial (RCT) examined the efficacy of restricted resuscitation fluid protocol, which could successfully reduce resuscitation fluid volumes in adult patients with septic shock^5)^. In this study, the standard care group received fluid boluses as long as circulation continued to show improvement, whereas the fluid restriction group received fluid boluses only when severe hypoperfusion appeared. However, the following larger RCT examining restricted intravenous fluid failed to show a better outcome^6)^. Thus, the effect of restricted fluid therapy is still unclear, but we think avoiding volume overload by regularly reassessing the patient’s response to fluid resuscitation is necessary.

Excess catecholamine use is toxic to the glycocalyx. The glycocalyx shedding is known to be markedly increased in Takotsubo disease, where the surge of catecholamine occurs^7)^. In cases of septic shock, when adequate fluid resuscitation fails to restore perfusion, the dose of vasopressors (norepinephrine) will be increased, and that can damage the vascular integrity. Therefore, Surviving Sepsis Campaign Guidelines 2021 state, “For adults with septic shock on norepinephrine with inadequate mean arterial pressure levels, we suggest adding vasopressin instead of escalating the dose of norepinephrine^8)^.” The effect of the restriction of catecholamine should be examined in the future study.

Hyperglycemia is also known to be related to glycocalyx injury. Nieuwdorp et al. reported that hyperglycemia increased plasma hyaluronan levels, endothelial dysfunction, and activation in coagulation^9)^. The mechanism can be explained by the increased reactive oxygen species and receptor activation for advanced glycation end-products (RAGE), but we demonstrated that neutrophil activation and neutrophil extracellular trap formation are also involved in the mechanisms of glycocalyx injury^10)^. Since hypoglycemia is harmful to septic patients, glycemic control by intensive insulin therapy is not the current trend in sepsis management^8)^. However, the toxic effect of hyperglycemia on the glycocalyx should be kept in mind.

Colloid therapy using fresh frozen plasma (FFP) and albumin has been proposed to protect glycocalyx. FFP contains physiological oxidase, protease, and matrix metalloproteinase inhibitors that help maintain the glycocalyx. Furthermore, FFP can suppress the fluid volume for resuscitation compared to crystalloids^11)^. Meanwhile, albumin is expected to protect glycocalyx via carrying erythrocyte-derived sphingosine-1-phosphate (S1P) to the endothelium, where it can mediate glycocalyx recovery by suppressing metalloproteinase activity^12)^. However, the result of the clinical trial was disappointing. A phase 2, multicenter randomized trial was conducted among the patients who underwent abdominal surgery. Dexamethasone and 20% albumin (100 mL) were followed by 200 mL of 20% albumin with subsequent 1000 mL of crystalloid administered to the study group, while crystalloid fluid was only given to the control group. The damage of the glycocalyx was compared by the levels of circulating syndecan-1, and the levels did not differ between the groups^13)^. However, the effects of colloid therapy should be reexamined in larger trials.

One of the pivotal functions of the glycocalyx is antithrombotic. Heparan sulfate is an essential glycosaminoglycan side-chain of the glycocalyx and is a cofactor of physiological anticoagulant antithrombin. Since antithrombin activity significantly decreases in sepsis, antithrombin supplementation is a rational approach for the management of sepsis-induced coagulopathy. It is of interest that antithrombin not only balances the hypercoagulation but can stabilize the glycocalyx by binding to the heparan sulfate^14)^.

Given the critical role the glycocalyx plays in maintaining vascular integrity, it is imperative to protect this component during sepsis management.

## Funding

No funding was received.

## Author contributions

JHL and TI wrote and reviewed the manuscript. Both authors read and approved the final manuscript.

## Conflicts of interest statement

The authors declare that they have not conflict of interest.
